# Evaluation of an Immunomodulatory Probiotic Intervention for Veterans With Co-occurring Mild Traumatic Brain Injury and Posttraumatic Stress Disorder: A Pilot Study

**DOI:** 10.3389/fneur.2020.01015

**Published:** 2020-10-20

**Authors:** Lisa A. Brenner, Jeri E. Forster, Kelly A. Stearns-Yoder, Christopher E. Stamper, Andrew J. Hoisington, Diana P. Brostow, Meredith Mealer, Hal S. Wortzel, Teodor T. Postolache, Christopher A. Lowry

**Affiliations:** ^1^VA Rocky Mountain Mental Illness Research Education and Clinical Center (MIRECC), Rocky Mountain Regional Veterans Affairs (VA) Medical Center (RMRVAMC), Aurora, CO, United States; ^2^Department of Physical Medicine and Rehabilitation, University of Colorado Anschutz Medical Campus, Aurora, CO, United States; ^3^Department of Psychiatry and Neurology, University of Colorado Anschutz Medical Campus, Aurora, CO, United States; ^4^Military and Veteran Microbiome: Consortium for Research and Education, Aurora, CO, United States; ^5^Department of Systems Engineering & Management, Air Force Institute of Technology, Wright-Patterson Air Force Base, OH, United States; ^6^Mood and Anxiety Program, University of Maryland School of Medicine, Baltimore, MD, United States; ^7^Veterans Integrated Service Network (VISN) 5 MIRECC, Department of Veterans Affairs, Baltimore, MD, United States; ^8^Department of Integrative Physiology, University of Colorado Boulder, Boulder, CO, United States; ^9^Center for Neuroscience, University of Colorado Boulder, Boulder, CO, United States; ^10^Center for Neuroscience, University of Colorado Anschutz Medical Campus, Aurora, CO, United States

**Keywords:** traumatic brain injury, posttraumatic stress disorder (PTSD), probiotic, Veteran, gut-brain axis, *Lactobacillus reuteri* DSM 17938, microbiome

## Abstract

**Background:** US military Veterans returned from Operation Enduring Freedom/Operation Iraqi Freedom/Operation New Dawn (OEF/OIF/OND) with symptoms associated with mild traumatic brain injury [mTBI; i.e., persistent post-concussive (PPC) symptoms] and posttraumatic stress disorder (PTSD). Interventions aimed at addressing symptoms associated with both physical and psychological stressors (e.g., PPC and PTSD symptoms) are needed. This study was conducted to assess the feasibility, acceptability, and safety of a probiotic intervention, as well as to begin the process of evaluating potential biological outcomes.

**Methods:** A pilot randomized controlled trial was implemented among US military Veterans from recent conflicts in Iraq and Afghanistan. Those enrolled had clinically significant PPC and PTSD symptoms. Participants were randomized to intervention (*Lactobacillus reuteri* DSM 17938) or placebo supplementation (daily for 8 weeks +/- 2 weeks) at a 1:1 ratio, stratified by irritable bowel syndrome status. Thirty-one Veterans were enrolled and randomized (15 to the placebo condition and 16 to the probiotic condition).

**Results:** Thresholds for feasibility, acceptability, and safety were met. Probiotic supplementation resulted in a decrease in plasma C-reactive protein (CRP) concentrations relative to the placebo group that approached statistical significance (*p* = 0.056). Although during the Trier Social Stress Test (TSST; administered post-supplementation) no between-group differences were found on a subjective measure of stress responsivity (Visual Analog Scale), there was a significantly larger increase in mean heart beats per minute between baseline and the math task for the placebo group as compared with the probiotic group (estimated mean change, probiotic 5.3 [95% Confidence Interval: −0.55, 11.0], placebo 16.9 [11.0, 22.7], *p* = 0.006).

**Conclusions:** Findings from this trial support the feasibility, acceptability, and safety of supplementation with an anti-inflammatory/immunoregulatory probiotic, *L. reuteri* DSM 17938, among Veterans with PPC and PTSD symptoms. Moreover, results suggest that CRP may be a viable inflammatory marker of interest. A larger randomized controlled trial aimed at measuring both biological and clinical outcomes is indicated.

**Clinical Trial Registration:** ClinicalTrials.gov, Identifier NCT02723344.

## Introduction

Many US Service Members who returned from Iraq and Afghanistan did so with co-occurring physical and mental health conditions, including mild traumatic brain injury (mTBI) and posttraumatic stress disorder (PTSD). It has been estimated that ~11–23% of those who served in Iraq or Afghanistan have a history of mTBI, with around 8% reporting persistent post-concussive (PPC) symptoms [e.g., sleep (insomnia), cognitive (memory loss, poor concentration, and problem solving); (1), emotional (depressed mood, irritability, and anxiety), and physical (headache, dizziness, exercise intolerance, fatigue, noise, and light sensitivity symptoms); (2)]. Among those with a history of mTBI and PPC symptoms, co-occurring psychiatric disorders (e.g., PTSD and depression) are common. According to work by Bahraini et al. (3) among military and Veteran samples with a history of TBI, “estimates of PTSD range from 12 to 89%” (p. 58). As such, there is ongoing discussion regarding the etiology of the non-specific symptoms associated with PPC syndrome, with a particular focus on whether such symptoms are more likely to be associated with physical (TBI) or psychological (PTSD) injury. For further discussion of the complicated relationship between TBI and PTSD, see Loignon et al. (4).

Despite their frequent co-occurrence, there is a dearth of evidence-based treatments that address symptoms of both conditions (3, 5). According to Hoge and Jonas “[t]he critical gap in clinical interventions will begin to get filled only when clinicians and researchers turn their attention to developing and validating interventions that respect the inherent non-specific multietiological nature of these symptoms…” (E2) (3, 5, 6). In addition, persistent symptoms associated with mTBI and PTSD are often resistant to traditional medical interventions (7). As such, “complementary and alternative medicine (CAM) may be sought by the patient…” [p. 116; (7)]. Over the past two decades, the use of CAM in the USA has seen a steady and steep rise (8–10). Findings from Betthauser et al. suggest that Veterans are open to, or are already using, CAM approaches (11). Interestingly, results also suggest that Veterans with symptoms associated with mTBI or PTSD may be more accepting of CAM approaches than those without these conditions (11).

Identification of interventions aimed at symptoms associated with both conditions would require addressing a common mechanism. Excessive neurologic and systemic inflammation has been found to play a role in the vulnerability to, and aggravation and perpetuation of, adverse consequences associated with both physical and psychological stressors (12–16). Following acute TBI in humans, cerebral inflammatory responses begin within minutes and include elevations of interleukin (IL)-1β, IL-6, IL-8, and tumor necrosis factor alpha (TNFα) (15). Post-injury (mild, moderate, and severe TBI) increases in cerebral inflammatory responses, including microglial and astroglial activation, can be prolonged, lasting months to years (15–17). For example, Johnson et al. found that neuroinflammatory processes in the corpus callosum can persist for many years after a single moderate-to-severe TBI (17). Among military personnel who sustained mTBIs during their last deployment, those with mTBI plus loss of consciousness had significantly elevated IL-6 concentrations compared to those with mTBI and no loss of consciousness or no history of TBI (16). Moreover, within both mTBI groups, increased TNFα was associated with greater PTSD symptoms (16). The influence of inflammatory responses on evolving symptomatology and pathology (17) underscores inflammation as a potential target for treatment, long after the acute physical or psychological trauma has taken place (13, 14, 16).

In prospective studies, high baseline plasma concentrations of the inflammatory marker, C-reactive protein (CRP), measured pre-deployment among military personnel, were associated with the increased likelihood of having PTSD symptoms post-deployment, thereby suggesting that inflammation prior to trauma exposure may predispose individuals to developing PTSD (18). Similarly, elevated inflammation within hours of a traumatic event may predict adverse psychological and neurological outcomes. For example, increased circulating levels of IL-6 are associated with increased trauma exposure and associated diagnoses [i.e., PTSD; (19)]. These clinical findings are supported by rodent studies demonstrating a causal role for elevated baseline IL-6 in vulnerability to an anxiety- and depression-like syndrome (20).

As noted above, pre-existing inflammation, as well as associated background immunodysregulation, are risk factors for the development of PPC and PTSD symptoms (15, 18). Pretreatment with an immunoregulation-inducing agent may be expected to attenuate development and persistence of symptoms. Bio-immunomodulatory probiotics, such as *Lactobacillus reuteri* DSM 17938, have the potential to decrease stress-induced inflammatory responses, while being highly accessible, low cost, self-sustaining (e.g., portable), and, based on previous safety and tolerability trials, without serious side effects. Due to their proven ability to bind to the pattern recognition receptor dendritic cell-specific intercellular adhesion molecule-3-grabbing non-integrin (DC-SIGN), to induce proliferation of regulatory T cells (Treg), and to increase production of anti-inflammatory cytokines, including IL-10 and transforming growth factor β (TGFβ), probiotics are logical candidates for treatment of co-occurring PPC and PTSD symptoms (21). Moreover, in pre-clinical models, administration of the immunoregulatory probiotic DSM 17938, *L. reuteri*, has been found to promote homeostasis in the gut microbiota (22, 23), alleviating dysbiosis and enhancing eradication of proinflammatory pathobionts, including *Helicobacter* species (22–26). *L. reuteri* has also been found to assist in restoring the intestinal mucosal barrier by increasing enterocyte migration, proliferation, and crypt height (22, 23, 27). Of note, restoration of the mucosal barrier reduces bacterial translocation to mesenteric lymph nodes, thereby further reducing systemic inflammation.

The following pilot trial of an immunomodulatory probiotic supplementation, *L. reuteri* DSM 17938, was implemented among Veterans from Operation Enduring Freedom/Operation Iraqi Freedom/Operation New Dawn (OEF/OIF/OND) with co-occurring PPC and PTSD symptoms using a longitudinal, double-blind, randomized placebo-controlled design. Study aims included evaluating the feasibility, acceptability, and safety of supplementation. Feasibility refers to the ease of implementation, and acceptability refers to the suitability of an intervention from the perspectives of the stakeholders [e.g., participants, and providers; (28)]. In addition, efforts were aimed at evaluating the impact of supplementation on biological markers (i.e., systemic inflammation, intestinal permeability, stress responsivity, and microbial diversity and composition).

## Materials and Methods

### Participants

This study was approved by the Colorado Multiple Institutional Review Board (IRB) and the local VA Research and Development Committee. Written informed consent was provided in accordance with the Declaration of Helsinki. Participants included US military Veterans between the ages of 18 and 50 with (1) at least one deployment in support of OEF/OIF/OND; (2) a history of mTBI per the Ohio State University (OSU) TBI-ID (29) with any endorsement of PPC symptoms associated with an mTBI that occurred at least 6 months prior to the baseline assessment; (3) current symptoms in three or more of the following International Classification of Diseases (ICD)-10 post-concussive (PC) symptom categories (30) as measured by the Rivermead Post-concussion Symptom Questionnaire [RPQ; score of 2 or greater per symptom to qualify as (a) headache, dizziness, malaise, fatigue, and noise intolerance; (b) irritability, depression, anxiety, and emotional lability; (c) subjective concentration, memory, or intellectual difficulties; and/or (d) insomnia] (31); (4) a current diagnosis of PTSD per the Clinician Administered PTSD Scale-5 (CAPS-5) (32); and (5) medical clearance by study providers. Exclusion criteria included: (1) history of moderate-to-severe TBI; (2) current involvement in the criminal justice system as a prisoner or ward of the state; (3) current (past month) alcohol or substance abuse or dependence; (4) lifetime history of bipolar disorder or psychosis or anxiety disorders; (5) consistent (e.g., 5×/week or greater) probiotic supplementation within the last month, including probiotic food products such as yogurt, as determined by phone screen interview and Probiotic Food Check List; (6) receiving antibiotics within the last month; (7) receiving medications that interfere with gut motility (opiates, loperamide, and stool softeners); (8) presence of central venous catheters (CVCs); (9) gastrointestinal (GI) barriers as identified by the 2-week run-in period as determined by the study team (e.g., daily GI discomfort with frequent diarrhea prior to supplementation); (10) participation in conflicting interventional research protocol; (11) vital signs outside of acceptable range, i.e., blood pressure >160/100, oral temperature >100°F, and pulse >100; (12) use of any drugs within the last 6 months [viz., systemic antibiotics, antifungals, antivirals, or antiparasitics (intravenous, intramuscular, or oral); oral, intravenous, intramuscular, nasal or inhaled corticosteroids; cytokines or cytokine inhibitors; methotrexate or immunosuppressive cytotoxic agents]; (13) acute disease at the time of enrollment; (14) chronic, clinically significant (unresolved, requiring on-going medical management or medication) pulmonary, cardiovascular, GI, hepatic, or renal functional abnormality, as determined by medical history or physical examination other than irritable bowel syndrome (IBS); (15) history of cancer except for squamous or basal cell carcinomas of the skin that have been medically managed by local excision; (16) unstable dietary history as defined by major changes in diet during the previous month, where the subject has eliminated or significantly increased a major food group in the diet; (17) positive test for human immunodeficiency virus (HIV), hepatitis B virus, or hepatitis C virus; (18) major surgery of the GI tract, with the exception of cholecystectomy and appendectomy, in the past 5 years or any major bowel resection at any time; (19) regular urinary incontinence necessitating use of incontinence protection garments; (20) female who is pregnant or lactating; (21) treatment for or suspicion of ever having had toxic shock syndrome; or (22) those receiving immunosuppressive drugs/medications (e.g., oral corticosteroids) or treatment including antineoplastic therapy, post-transplantation immunosuppressive therapy, and/or radiation therapy.

Participant demographics and clinical characteristics [e.g., IBS status as measured by the Rome III Diagnostic Questionnaire (33) and dietary diversity] are presented in Table 1. The average age of the male Veterans included in the study was 37.4 years (SD 6.7). The sample was 68% Caucasian. No significant demographic or military characteristic differences were noted. In terms of most recent and worst mTBIs (lifetime), there were no significant between-group differences in terms of the number of participants in each group with alterations of consciousness (AOC) vs. losses of consciousness (LOC) [most recent: AOC—placebo 8 (53%), probiotic 13 (81%), LOC—7 (47%), 3 (19%), *p* = 0.14; worst: AOC—placebo 4 (27%), probiotic 9 (56%), LOC—placebo 11 (73%), probiotic 7 (44%), *p* = 0.10]. There were also no between group differences in terms of reported specific lengths of LOC (most recent/worst mTBI) reported among those with such a loss (*p* = 0.53, *p* = 0.38). Though not significant, 69% of those in the probiotic group had “some college” compared with 33% in the placebo group. No significant differences were noted on clinical interviews/participant reported outcomes at baseline regarding PPC symptoms, PTSD symptoms, sleep, or diet.

**Table 1:** Participant demographics and clinical characteristics.

	**Total (*N* = 31)**	**Placebo (*N* = 15)**	**Probiotic (*N* = 16)**	***P*-value**
**Demographics**				
Age(*years*)^a^	37.4 (6.7) 36 (26, 50)	36.7 (6.2) 36 (28, 50)	37.9 (38.5) 38.5 (26, 50)	0.65
Male^b^	31 (100%)	15 (100%)	16 (100%)	>0.99
Race^b^				
Caucasian	21 (68%)	10 (67%)	11 (69%)	0.56
African American	2 (6%)	1 (7%)	1 (6%)	
Native American/Alaskan Native	2 (6%)	2 (13%)	0 (0%)	
Multiracial	4 (13%)	2 (13%)	2 (12.5%)	
Other	2 (6%)	0 (0%)	2 (12.5%)	
Hispanic^b^	3 (10%)	2 (15%)	1 (6%)	0.59
Education^b^				
Some college	16 (52%)	5 (33%)	11 (69%)	0.06
Associate's	7 (23%)	5 (33%)	2 (29%)	
Bachelor's	6 (19%)	5 (33%)	1 (6%)	
Master's	1 (3%)	0 (0%)	1 (6%)	
Doctoral	1 (3%)	0 (0%)	1 (6%)	
Marital Status^b^				
Married	21 (68%)	11 (73%)	10 (48%)	0.76
Single	4 (13%)	1 (7%)	3 (19%)	
Cohabitating	3 (10%)	2 (13%)	1 (6%)	
Divorced/Separated	3 (10%)	1 (7%)	2 (13%)	
Sexual Orientation^b^				
Heterosexual	28 (90%)	14 (93%)	14 (88%)	>0.99
Gay/Lesbian/Queer	1 (3%)	1 (7%)	0 (0%)	
Bisexual	1 (3%)	0 (0%)	1 (6%)	
Other	1 (3%)	0 (0%)	1 (6%)	
Employment^b^				
Full time	18 (58%)	9 (60%)	9 (56%)	>0.99
Part time	3 (10%)	1 (7%)	2 (13%)	
Unemployed/Not seeking	3 (10%)	1 (7%)	2 (13%)	
Unemployed/Seeking	3 (10%)	2 (13%)	1 (6%)	
Retired	4 (13%)	2 (13%)	2 (13%)	
Student^b^				
Full time	10 (32%)	6 (40%)	4 (25%)	0.15
Part time	2 (6%)	2 (13%)	0 (0%)	
No	19 (61%)	7 (47%)	12 (75%)	
Homeless^b^	0 (0%)	0 (0%)	0 (0%)	>0.99
Military Branch^b^				
Army	27 (87%)	14 (93%)	13 (81%)	0.60
Air Force	1 (3%)	0 (0%)	1 (6%)	>0.99
Navy	3 (10%)	1 (7%)	2 (13%)	>0.99
Marines	2 (6%)	1 (7%)	1 (6%)	>0.99
Era^b^				
Post-Vietnam	2 (6%)	1 (7%)	1 (6%)	>0.99
Desert Storm	6 (19%)	2 (13%)	4 (25%)	0.65
OEF/OIF/OND	31 (100%)	15 (100%)	16 (100%)	>0.99
Months active duty^a^	108 (68) 95 (12, 264)	111 (68) 96 (18, 264)	106 (69) 83.5 (12, 224)	0.97
Number of deployments^a^	2.6 (1.5) 2 (1, 7)	2.4 (1.5) 2 (1, 6)	2.8 (1.6) 2.5 (1, 7)	0.38
Number of combat tours^a^	2 (1.4) 2 (0, 6)	2 (1.5) 2 (0, 6)	2 (1.4) 2 (0, 5)	0.90
**Clinical characteristics**				
Irritable Bowel Syndrome (per Rome III Diagnostic Questionnaire^b^)	7 (23%)	3 (20%)	4 (25%)	>0.99
Ohio State University TBI-ID				
Time since most recent mTBI (years)^a^	10.5 (7.1) 10 (1, 33)	8.3 (3.4) 9 (1, 12)	12.4 (9.1) 12 (1, 33)	0.14
Number of lifetime mTBIs^a^	2.7 (1.5) 2 (1, 6)	3.3 (1.7) 3 (1, 6)	2.1 (1.1) 2 (1, 4)	0.05
The Clinician Administered PTSD Scale-5 (CAPS-5)^a^				
Total severity	34.5 (10.2) 32 (16, 59)	34 (10.5) 38 (16, 49)	35.1 (10.1) 32 (22, 59)	0.72
Criterion B severity (Re-experiencing)	8.3 (3.0) 8 (3, 14)	8.6 (3.0) 8 (3, 14)	8.0 (3.0) 7.5 (4, 14)	0.47
Criterion C severity (Avoidance)	4.0 (1.6) 4 (2, 6)	4.0 (1.6) 4 (2, 6)	4.1 (1.7) 4.5 (2, 6)	0.90
Criterion D severity (Negative alterations in cognitions and mood)	11.6 (4.9) 11 (4, 23)	10.6 (5.2) 9 (4, 20)	12.5 (4.7) 12 (6, 23)	0.23
Criterion E severity (Alterations in arousal and reactivity)	10.6 (3.3) 10 (6, 17)	10.8 (3.4) 11 (6, 15)	10.5 (3.2) 10 (7, 17)	0.69
Neurobehavioral Symptom Inventory (NSI)^a^				
Cognitive	8.0 (4.6) 8 (0, 16)	8.4 (4.7) 9 (1, 16)	7.7 (4.6) 7.5 (0, 14)	0.72
Mood-behavioral	14.5 (6.5) 15 (4, 27)	13.7 (6.7) 15 (4, 26)	15.2 (6.5) 14.5 (5, 27)	0.63
Vestibular-sensory	12.6 (7.6) 13 (0, 27)	11.5 (7.6) 10 (0, 24)	13.6 (7.8) 14 (1, 27)	0.59
**Pittsburgh Sleep Quality Index (PSQI)^a^^,^^c^**	**Total (*****N*** **=** **28)**	**Placebo (*****N*** **=** **14)**	**Probiotic (*****N*** **=** **14)**	***p*-value**
Duration (0–3)	1.61 (1.2) 2 (0, 3)	1.36 (0.3) 1 (0, 3)	1.86 (1.1) 2 (0, 3)	0.33
Sleep disturbance (0–3)	1.85 (0.66)^d^ 2 (1, 3)	1.93 (0.73) 2 (1, 3)	1.77 (0.60)^e^ 2 (1, 3)	0.69
Sleep latency (0–3)	2.14 (1.0) 2.5 (0, 3)	2.21 (0.89) 2 (0, 3)	2.07 (1.2) 3 (0, 3)	>0.99
Day dysfunction (0–3)	1.46 (0.64) 1 (0, 3)	1.43 (0.76) 1 (0, 3)	1.50 (0.52) 1.5 (1, 2)	0.74
Sleep efficiency (0–3)	0.96 (1.3) 0 (0, 3)	1.29 (1.4) 0.5 (0, 3)	0.64 (1.0) 0 (0, 3)	0.27
Overall sleep quality (0–3)	1.96 (0.74) 2 (1, 3)	1.86 (0.77) 2 (1, 3)	2.07 (0.73) 2 (1, 3)	0.51
Need meds to sleep (0–3)	1.36 (1.5) 0.5 (0, 3)	1.07 (1.5) 0 (0, 3)	1.64 (1.4) 2.0 (0, 3)	0.26
PSQI total (0–21)	11.4 (4.5)^d^ 13 (4, 18)	11.1 (5.3) 10 (4, 18)	11.8 (3.5)^e^ 13 (6, 16)	0.88
**Dietary Diversity Score (DDS)^a^^,^^f^**	**Total (*****N*** **=** **29)**	**Placebo (*****N*** **=** **13)**	**Probiotic (*****N*** **=** **16)**	***p*-value**
Total^g^	0.66 (0.12) 0.69 (0.30, 0.91)	0.68 (0.1) 0.69 (0.51, 0.80)	0.65 (0.14) 0.66 (0.30, 0.91)	0.48
Fruits and vegetables^h^	0.7 (0.18) 0.74 (0.37, 0.94)	0.72 (0.18) 0.74 (0.37, 0.94)	0.68 (0.19) 0.71 (0.37, 0.91)	0.63
Complex carbohydrates^i^	0.56 (0.19) 0.54 (0.23, 1.0)	0.54 (0.2) 0.46 (0.23, 0.92)	0.57 (0.19) 0.54 (0.23, 1.0)	0.52
Animal foods^j^	0.62 (0.18) 0.66 (0.09, 0.94)	0.66 (0.16) 0.69 (0.34, 0.94)	0.59 (0.19) 0.59 (0.09, 0.88)	0.22
Plant proteins^k^	0.67 (0.18) 0.67 (0.22, 1.0)	0.67 (0.13) 0.67 (0.44, 0.89)	0.67 (0.22) 0.67 (0.22, 1.0)	0.89

a *Mean (SD) and Median (range)*.

b *N (%) is presented*.

c *Higher PSQI scores indicate worse sleep quality*.

d *N = 27*.

e *N = 13*.

f *DDS is calculated as the number of foods eaten on a regular basis as a proportion of all possible foods*.

g *Total DDS comprises regularly eaten foods as a proportion of 144 possible foods included on the Harvard Food Frequency Questionnaire*.

h *Fruits and Vegetables comprises 35 possible foods, including a range of fruits and vegetables such as stone fruits, apples, berries, melons, citrus, avocado, cruciferous vegetables, alliums, root vegetables, and tomatoes, among others*.

i *Complex Carbohydrates comprises 13 possible foods: oatmeal, cooked grain cereals, rye bread, dark bread, brown rice and other grains, corn, popcorn, oat bran, other bran, wheat germ, potatoes, and sweet potatoes*.

j *Animal Foods comprises 32 possible foods of animal origin, including milk, fermented dairy products, eggs, red meat, poultry, fish and seafood, and processed meat products (e.g., hot dogs)*.

k *Plant Proteins comprises 9 foods that are commonly consumed as plant-based sources of protein: tofu, string beans, peas and lentils, beans, peanuts and peanut butter, walnuts, other tree nuts, and soy milk*.

### Randomization

Processes were in place to randomize to intervention or placebo at a 1:1 ratio, stratified by gender and IBS, as measured by the Rome III Diagnostic Questionnaire (33). Despite efforts to recruit both women and men, no female Veterans met inclusion criteria. The decision to stratify by IBS was related to the findings by Maguen et al. (34) regarding the association between GI disorders (GIDs) and mental health conditions among OEF/OIF Veterans. Moreover, in conceptualizing PTSD as a more holistic disorder, there was concern that those with IBS may be more responsive to a probiotic intervention than those without GIDs.

### Study Procedures

**Figure 1:**
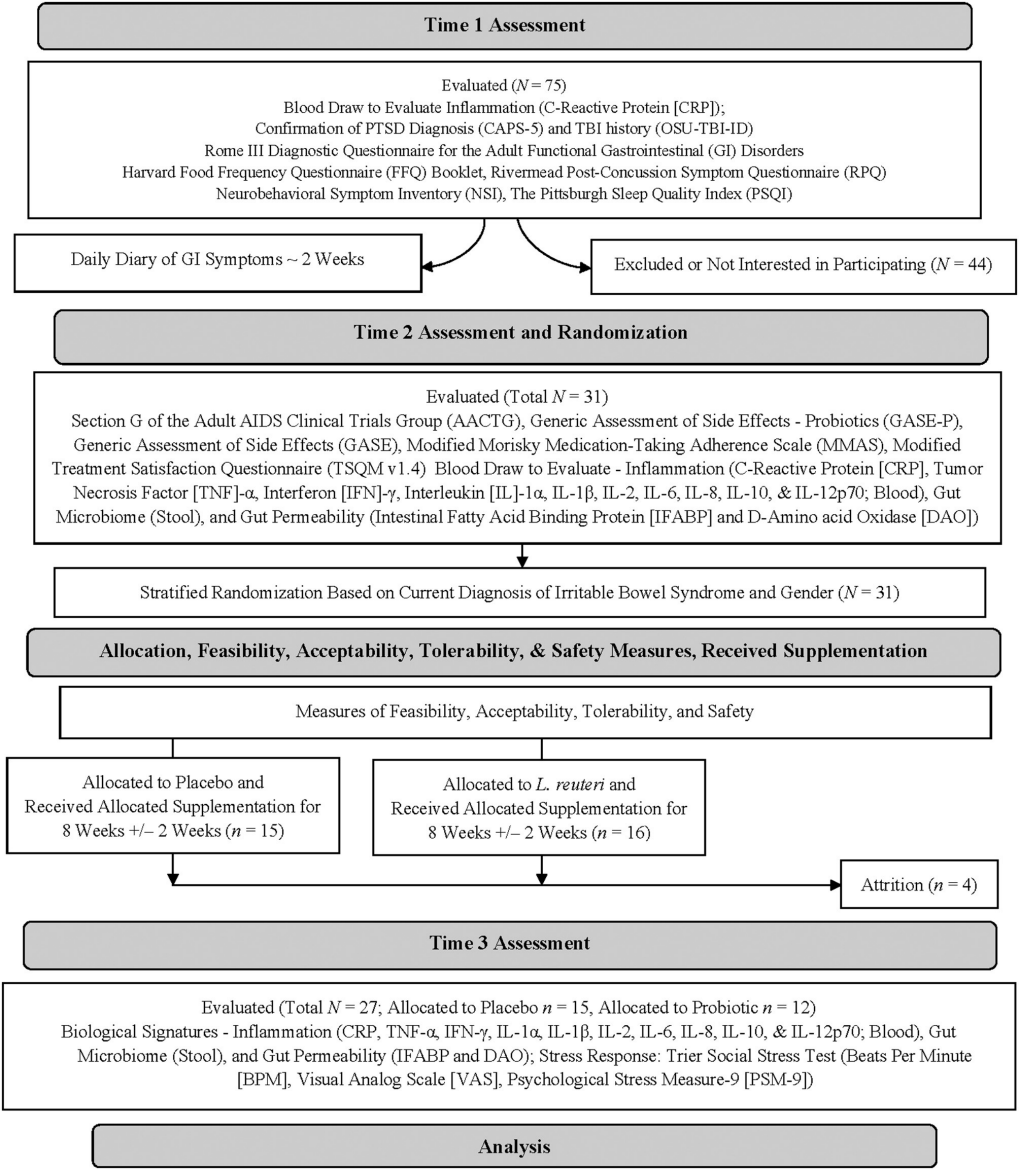
CONSORT diagram depicting the study design.

Study data were acquired over multiple timepoints (see Figure 1, CONSORT diagram). Fecal microbiome samples were collected pre- (Time 2, Assessment and Randomization) and post-supplementation (Time 3, Assessment) by self-sampling. Participants were provided with kits that included one BBL^®^ CultureSwab^®^ EZ Sterile double-tipped swab (BD, Franklin Lakes, NJ, USA), a sealable tube, and one pair of latex gloves to collect a stool sample. Blood samples were collected pre- (Time 2, Assessment and Randomization) and post-supplementation (Time 3, Assessment) for quantification of plasma concentrations of biomarkers of inflammation and gut permeability. The Trier Social Stress Test (TSST) was administered during the Time 3 Assessment.

### Intervention

All participants were randomized to receive either *L. reuteri* DSM 17938 drops, 100 million colony-forming units (CFU) in sunflower and medium chain triglyceride oils or placebo (sunflower and medium chain triglyceride oils) daily for 8 ± 2 weeks. *L. reuteri* DSM 17938 [deposited in the Deutsche Sammlung von Mikroorganismen und Zellkulturen (DSMZ) and referenced as DSM 17938; Gerber Soothe Colic Drops, 100 million CFU/5 drops; derived from *L. reuteri* American Type Culture Collection (ATCC) 55730] was used in the study.

### Measures

#### Baseline Demographics and Mental/Physical Health Characteristics

Multiple measures were used to assess participants' baseline mental and physical health. The CAPS-5 is a gold-standard structured clinical interview used to determine PTSD diagnosis (32). The Neurobehavioral Symptom Inventory (NSI) is a measure of PPC symptoms that is widely used among military personnel and Veterans and is recommended for screening and evaluation (35). Finally, the OSU-TBI-ID is a structured clinical interview to assess for history of TBI (29). Additional baseline clinical information was collected from each participant. The Harvard Food Frequency Questionnaire (FFQ) booklet is a comprehensive 101-item semi-quantitative FFQ that includes questions on the intake of specific foods and supplements and on adherence to particular diet types (36). Analysis of the questionnaire provides a wide range of macro- and micronutrient quantities. The Pittsburgh Sleep Quality Index (PSQI) was used to measure a range of factors associated with sleep (e.g., duration, frequency of disturbances, and overall sleep quality) (37, 38). The Rome III Diagnostic Questionnaire for the Adult Functional Gastrointestinal Disorders classifies disorders of the digestive system (33). This comprehensive questionnaire takes 20–30 min to complete and has been used to identify inclusion criteria for clinical research studies (33).

#### Feasibility and Acceptability

Participants were provided with a probiotics usage log, one of several assessment measures linked to feasibility (28). The participants documented the date, time, and amount of each probiotic dose taken, as well as any symptoms experienced and their severity. The document was modeled after the Dana-Farber Harvard Cancer Center (DF/HCC) Oral Chemotherapy Drug Diary. In addition, section G of the Adult AIDS Clinical Trials Group (AACTG) (39) is a 14-item self-report questionnaire administered to query why one might not take a supplement as directed. The Generic Assessment of Side Effects (GASE)—Probiotics (GASE-P) is identical to GASE but assesses side effects related to supplements (40). The GASE consists of 36 symptom descriptions organized by parts of the body. Participants were asked to rate if these “symptoms” were “not present,” “mild,” “moderate,” or “severe” in the past week related to current medication. The Modified Morisky Medication-Taking Adherence Scale (MMAS) is an eight-item self-report questionnaire designed to test whether or not a subject adheres to taking a particular medication or supplement and has demonstrated concurrent and predictive validity, in regard to the measurement of patient adherence in clinical studies (41, 42). If a participant scores higher on the scale, they are more adherent to the supplement. The Modified Treatment Satisfaction Questionnaire (TSQM v1.4) is a nine-item valid questionnaire that is designed to measure side effects associated with the supplement itself and the ease of administration of the supplement (43). Participant retention and adverse event records were maintained by study staff.

#### Biomarkers of Inflammation

Converging lines of evidence have shown that inflammation is associated with mTBI and the development of PTSD (12–16). Therefore, we measured multiple systemic biomarkers of inflammation from plasma. These consisted of CRP, measured by high-sensitivity enzyme-linked immunosorbent assay (ELISA) (Cat No. DCRP00, R&D Systems, Minneapolis, MN, USA), and a panel of cytokines [IL-1α, IL-1β, IL-2, IL-6, IL-8, IL-10, IL-12p70, TNFα (active trimer), and interferon gamma (IFNγ)] measured by high-sensitivity multiplex ELISA (Cat No. 85-0002, Quanterix, Billerica, MA, USA).

#### Intestinal Permeability

Biomarkers of intestinal permeability consisted of intestinal fatty acid binding protein (IFABP) (Cat No. DFBP20, R&D Systems) and d-amino acid oxidase (DAO) (Cat No. SEJ298Hu, Cloud Clone, Katy, TX, USA) measured by ELISA. All assays were run according to the manufacturer's instruction manuals.

#### Stress Responsivity

To evaluate stress responsivity, the TSST (44, 45) was administered. The TSST is a standard laboratory assessment that has been used in a variety of populations, including military personnel and individuals with mental health conditions (46). With the use of a remote heart rate monitor placed around the base of the sternum, heart beats per minutes (BPM) was continuously measured during the TSST. Signals were recorded with an ADInstruments system. Timepoints of interest for BPM were at baseline (20-min relaxation), pre-speech, during the speech, during the math task, and after a period of relaxation (10-min post-relaxation). In the speech task, participants are asked to give a 5-min presentation on why they are the best candidate for a job. During the presentation, evaluators maintain neutral expressions. If the participant does not present for the entire 5 min, they are asked to continue until time has expired. In the math task, participants are asked to count backwards by 13s. If they make a mistake, they have to start from the original number given. The Visual Analog Scale (VAS; distress thermometer) is a psychometric response scale for subjective characteristics or attitudes (47). Participants responded to their subjective level of stress on a 0–10 Likert scale. The VAS was administered before the speech task, immediately after the speech task, and immediately after the math task.

#### Microbiome

Sample DNA was extracted using the PowerSoil DNA extraction kit (Cat No. 12955-4, Qiagen, Valencia, CA, USA) according to the manufacturer's instructions. Marker genes in isolated DNA were polymerase chain reaction (PCR) amplified using GoTaq Master Mix (Cat No. M5133, Promega, Madison, WI, USA); 515 F (5′-GTGCCAGCMGCCGCGGTAA-3′) and 806 R (5′-GGACTACHVGGGTWTCTAAT-3′) primer pair (Integrated DNA Technologies, Coralville, IA, USA) targeting the V4 hypervariable region of the 16S rRNA gene modified with a unique 12-base sequence identifier for each sample; and the Illumina adapter as previously described in Caporaso et al. (48) The thermal cycling program consisted of an initial step at 94°C for 3 min followed by 35 cycles (94°C for 45 s, 55°C for 1 min, and 72°C for 1.5 min), and a final extension at 72°C for 10 min. PCRs were run in duplicate, and the products from the duplicate reactions were pooled and visualized on an agarose gel to ensure successful amplification. PCR products were cleaned and normalized using a SequalPrep Normalization Kit (Cat. No. A1051001, ThermoFisher, Waltham, MA, USA) following the manufacturer's instructions. The normalized amplicon pool was sequenced on an Illumina MiSeq run by using V3 chemistry and 600 cycles, 2 × 300-bp paired-end sequencing. All library preparation and sequencing were conducted at the University of Colorado Boulder BioFrontiers Next-Gen Sequencing core facility, https://bficores.colorado.edu/sequencing-lab.

### Analyses

#### Baseline Demographics and Mental/Physical Health Characteristics

Demographic and clinical characteristics were compared between groups using Wilcoxon rank-sum and Fisher's exact tests, as appropriate.

#### Feasibility and Acceptability

Measures are reported as both means and standard deviations, as well as medians and ranges, and were compared between groups using Wilcoxon rank-sum tests. All analyses were run in SAS v9.4 and R v3.5.3. In terms of feasibility, we used the criteria of Thabane et al. (49) who suggest classifying outcomes as follows: “(i) Stop—main study not feasible; (ii) Continue, but modify protocol—feasible with modifications; (iii) Continue without modifications, but monitor closely—feasible with close monitoring; and (iv) Continue without modifications—feasible as is.” The *a priori* criterion for continuing without modifications (feasible as is) was as follows: medium adherence with supplementation in at least 50% of all participants on all administrations of the MMAS and retention of 50% through at least six such administrations.

#### Stress Responsivity

A mixed-effects model with a random intercept was used to investigate heart BPM during the TSST (baseline, pre-speech, speech, math, and post-relax), representing repeated measures on a participant over time. Within this model, each group was allowed to have its own trajectory, and a natural cubic B-spline transformation was performed on the timepoints to allow for smoothly varying trajectories. A search was performed to arrive at the overall best model, using a range of 1–3 degrees of freedom (df) for each group (all combinations were investigated for a total of nine models). Akaike's information criterion (AIC) was used to select the final model. A contrast within the final model was used to compare the change from baseline to math between the groups. A similar analysis was used to investigate changes in perceived stress (VAS), though these were collected at three TSST timepoints; a smaller range of 1–2 df was allowed for each group, and a total of four models were run for each outcome. AIC was again used to select the final models. Estimates from the VAS are reported, and the change from baseline to after math was compared between groups for the VAS using a contrast within the model.

#### Biomarkers of Inflammation and Intestinal Permeability

Given small sample sizes, differences in 8-week changes for plasma concentrations of CRP, plasma concentrations of cytokines, and plasma concentrations of biomarkers of intestinal permeability measures were compared using Wilcoxon rank-sum tests.

#### Probiotic and Microbiome

Sequencing data were processed and analyzed using Quantitative Insights Into Microbial Ecology (QIIME2 v. 2019.10) (50). The DADA2 algorithm was used to denoise demultiplexed sequences (51). Quality-filtered sequences were assigned taxonomic classification based on the SILVA 132 database released on December 2017 (52). Further analyses of α-diversity, β-diversity, and taxonomic composition of microbiomes were performed with QIIME2 v. 2019.10 and the open source statistical package R v. 3.5.1 (https://www.R-project.org). For α-diversity, β-diversity, and taxonomic composition of microbiomes, samples were rarefied to 11,900 sequences per sample. α-Diversity metrics included (1) observed operational taxonomic units (OTUs); (2) Shannon diversity; and (3) Faith's phylogenetic diversity (Faith's PD). Kruskal–Wallis test was used to examine for differences among treatment group and timepoint. For β-diversity, permutational multivariate analysis of variance (PERMANOVA) was performed using the vegan package (53) with the following distance metrics: unweighted UniFrac and weighted UniFrac. Analysis of Composition of Microbiomes (ANCOM) (54) was used to determine differentially abundant taxa at the phylum and genus levels.

## Results

Overall, 75 individuals were screened, and 31 Veterans were enrolled and randomized (15 to the placebo condition and 16 to the probiotic condition).

### Feasibility

Findings from the MMAS (range 0–8) suggested that both groups were highly adherent across all study weeks (placebo median = 7.6; probiotic median = 7.3), thereby suggesting feasibility in terms of supplementation adherence. This was further supported by findings from the AACTG (39) adherence questionnaire. The most frequent reason reported in both groups for not taking supplementation was forgetting. Nonetheless, participants in both groups reported “rarely” forgetting to take the supplement (placebo mean = 0.11; probiotic mean = 0.16).

### Acceptability

Based on scores from the TSQM, both groups (placebo and probiotic) found the supplementation acceptable. No differences were noted between groups in terms of side effects, convenience, or global satisfaction. Time 3 global satisfaction scores were ~70 (out of 100) for both groups, thereby suggesting the acceptability of *L. reuteri* DSM 17938 supplementation for those with chronic PPC and PTSD symptoms.

On the GASE-P, no differences were noted between groups in terms of probiotic-specific side effects or the severity of symptoms. Interestingly, when participants were asked to rate if these “symptoms” were “not present,” “mild,” “moderate,” or “severe” in the past week related to current medication, side effects notably decreased for both groups over time (see Table 2). In addition, no severe study-related adverse events were reported throughout the trial.

**Table 2:** Generic Assessment of Side Effects-Probiotics (GASE-P) scores by timepoint.

	**Full Sample**		**Placebo**		**Probiotic**		***p*-value**
	***N***	**Mean (SD) Median (range)**	***N***	**Mean (SD) Median (range)**	***N***	**Mean (SD) Median (range)**
**Time 1**							
Severity	31	26.9 (13.1) 25 (6, 48)	15	25.6 (13.9) 25 (6, 48)	16	28.1 (12.7) 25 (8, 47)	0.60
Symptom count	31	15.2 (5.9) 16 (5, 28)	15	14.4 (5.5) 16 (5, 22)	16	15.9 (6.4) 15 (5, 28)	0.65
**Time 2**							
Severity	29	22.9 (11.1) 24 (6, 52)	15	22.1 (13.2) 24 (6, 52)	14	23.7 (8.8) 22.5 (11, 41)	0.58
Symptom count	29	13.9 (5.8) 15 (4, 24)	15	12.4 (6.4) 14 (4, 24)	14	15.6 (4.8) 15 (9, 24)	0.19
**Time 3**							
Severity	27	12.9 (11.5) 8 (0, 39)	15	11.7 (11.3) 8 (0, 39)	12	14.3 (12.0) 12.5 (0, 33)	0.52
Symptom Count	27	8.0 (6.7) 7.0 (0, 29)	15	6.5 (4.5) 5 (0, 16)	12	9.8 (8.5) 7.5 (0, 29)	0.31

### Inflammatory Markers

Probiotic supplementation resulted in a decrease in plasma CRP concentrations that approached statistical significance. See Table 3 for results.

**Table 3:** Mean change in plasma concentrations of C-reactive protein (CRP), plasma concentrations of cytokines and plasma concentrations of biomarkers of intestinal permeability from Visit 2 to Visit 3.

	**Placebo**		**Probiotic**		***p*-value**
	***N***	**Mean (SD) and Median (range)^a^**	***N***	**Mean (SD) and Median (range)^a^**	
**Biomarkers of Inflammation**					
CRP (mg/L)	14	0.113 (1.2) 0.09 (−2.3, 2.4)	10	−0.625 (1.1) −0.565 (−2.3, 1.5)	0.056
IFNγ (pg/ml)	14	0.36 (1.2) 0.04 (−1.4, 3.9)	9	−0.33 (1.2) −0.04 (−3.1, 1.1)	0.22
IL1α (pg/ml)	14	0.98 (4.0) 0.28 (−4.4, 12.7)	9	−1.6 (11.2) 1.64 (−30.4, 9.6)	0.45
IL1β (pg/ml)	14	0.70 (2.7) 0.04 (−3.5, 9.1)	9	−0.25 (2.5) −0.03 (−6.1, 3.3)	0.37
IL2 (pg/ml)	14	0.02 (0.34) −0.02 (−0.60, 0.81)	9	−0.12 (1.3) 0 (−3.4, 1.3)	0.51
IL6 (pg/ml)	14	0.22 (2.4) −0.10 (−5.1, 4.0)	9	−0.07 (7.7) −0.24 (−13.5, 16.3)	0.64
IL8 (pg/ml)	14	−4.8 (11.2) −0.49 (−39.2, 3.6)	9	1.4 (5.9) 1.1 (−8.8, 11.4)	0.12
IL10 (pg/ml)	14	0.15 (0.85) 0.09 (−1.4, 2.2)	9	−0.23 (2.0) −0.23 (−3.0, 2.4)	0.72
IL12p70 (pg/ml)	14	−1.6 (9.3) −0.41 (−28.5, 11.2)	9	−10.1 (46.2) −0.61 (−129, 34.3)	0.89
IL-6:IL-10 ratio	14	−0.08 (1.0) −0.004 (−3.2, 1.6)	9	−0.003 (0.97) −0.12 (−0.7, 1.6)	0.52
TNFα (pg/ml)	14	−0.62 (1.4) −0.27 (−3.7, 1.3)	9	−2.1 (8.2) 0 (−23.7, 3.2)	0.26
**Intestinal Permeability**					
DAO (ng/ml)	10	−0.11 (0.24) −0.08 (−0.55, 0.29)	8	0.03 (0.12) 0.08 (−0.12, 0.16)	0.17
IFABP (pg/ml)	12	−0.09 (0.45) −0.17 (−1.0, 0.72)	10	−0.04 (0.16) −0.03 (−0.35, 0.21)	0.54

a *Negative numbers indicate a decrease from Visit 2 to Visit 3. Wilcoxon rank-sum test was used to compare the change from Visit 2 to Visit 3 between the Placebo and Probiotic groups. CRP, C-reactive protein; DAO, D-amino acid oxidase; IFABP, intestinal fatty acid binding protein; IFNγ, interferon gamma; IL, interleukin; mg/L, milligrams/liter; ng/ml, nanograms/ milliliter; pg/ml, picograms/ milliliter; TNFα, tumor necrosis factor alpha*.

### Intestinal Permeability

No differences in outcomes were noted between groups (see Table 3).

### Stress Responsivity

**Figure 2:**
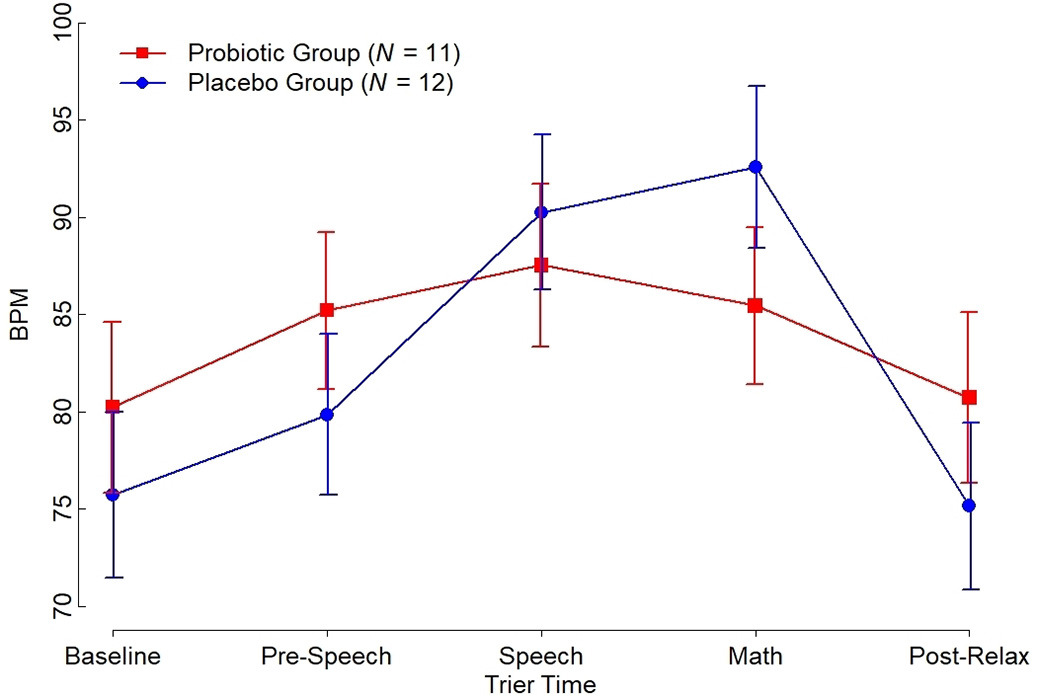
Beats per Minute (BPM) with Standard Error Bars by Trier Social Stress Test.

Both objective and subjective measures of stress were obtained. Participants in both the placebo and supplement groups experienced an increase in estimated perceived stress using the VAS over the course of the TSST (estimated mean, probiotic [*n* = 12]: baseline, 5.38 [95% CI: 3.85, 6.90], after speech 6.25 [95% CI: 4.87, 7.63], after math 7.13 [95% CI: 5.60, 8.65]; placebo [*n* = 15]: baseline 4.13 [95% CI: 2.77, 5.50], after speech 5.29 [95% CI: 4.04, 6.54], after math 6.44 [95% CI: 5.06, 7.84]). Moreover, no significant between-group differences were noted on estimated changes in VAS scores from baseline to after math (estimated change, probiotic 1.75 [95% CI: 0.49, 3.01]; placebo 2.31 [95% CI: 1.15, 3.47], *p* = 0.51). Nonetheless, during the TSST, there was a significantly larger increase in mean BPM between baseline and math for the placebo group as compared with the probiotic group (estimated change, probiotic 5.3 [95% CI: −0.55, 11.0], placebo 16.9 [95% CI: 11.0, 22.7], *p* = 0.006). Of note, the groups were not significantly different at baseline (estimated difference at baseline, 4.5 [95% CI: −7.66, 16.7], *p* = 0.46; see Figure 2).

### Microbiome

#### Presence of *L. reuteri* DSM 17938 in the Supplement

The presence of *L. reuteri* DSM 17938 in the prepared supplement was confirmed through amplification with species-specific primers using SYBR Green master mix in a Bio-Rad CFX qPCR machine. Positive placebo for the amplification was measured with *L. reuteri* DSM 17938 DNA provided by ATCC and compared with DNA extracted from three separate probiotic oil samples. DNA for the positive placebo and three samples was quantified for four dilution factors of 1, 1:10, 1:100, and 1:1,000. Results showed that the positive placebo and all three samples amplified at each of the four dilution factors. All the melt curves indicated amplification of the same target sequence, with no indication of non-specific amplification. The highest dilution factor (1:1,000) had CT values still under 30 for the probiotic samples. In addition, the three probiotic samples had similar amplification in each of the four dilution factors, indicating nearly uniform *L. reuteri* DSM 17938 concentrations in the probiotic samples.

#### α-Diversity, β-Diversity, and Taxonomic Composition of the Gut Microbiome

**Figure 3:**
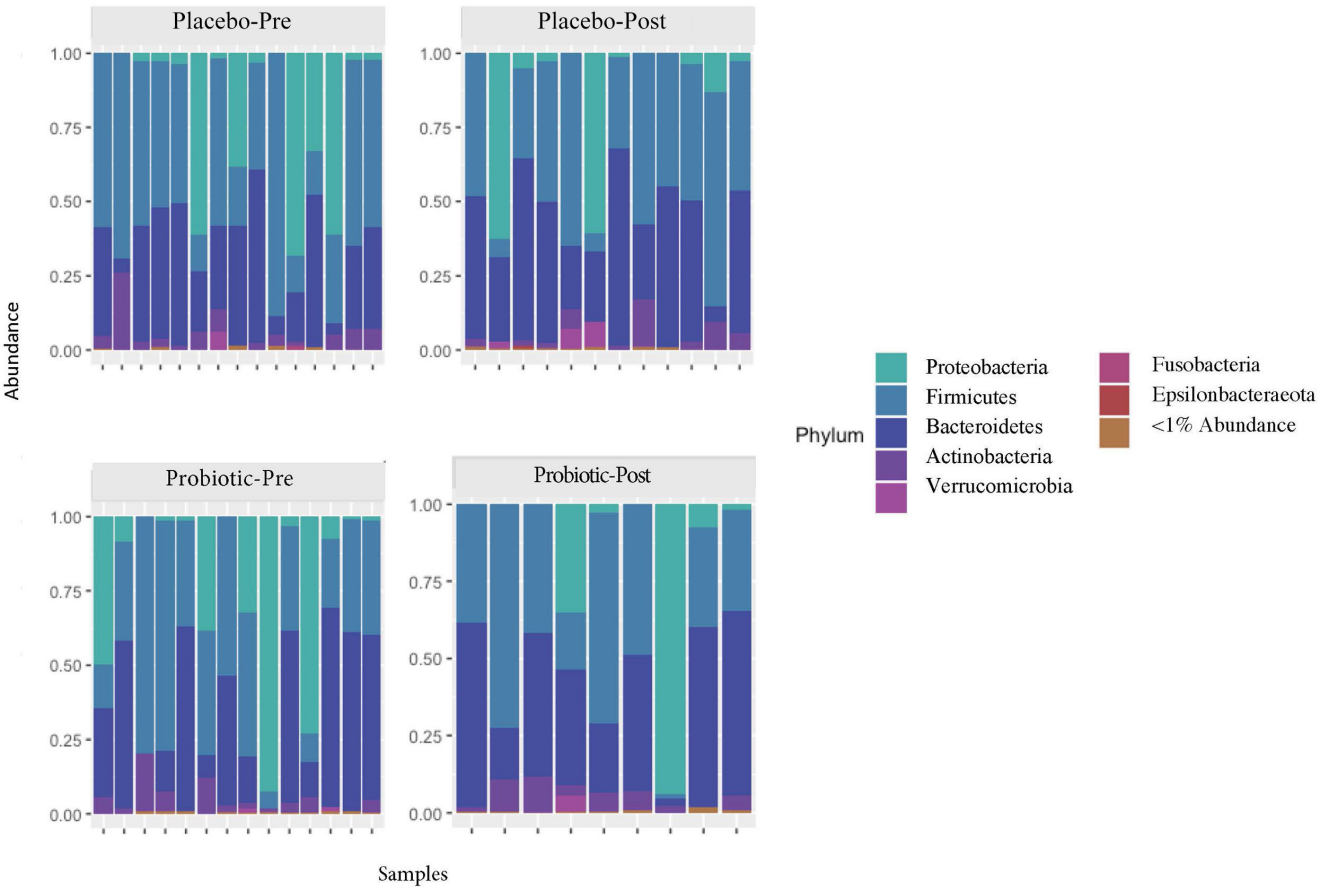
Relative abundances of phyla in the participant gut microbiome, before and after placebo or probiotic administration.

**Figure 4:**
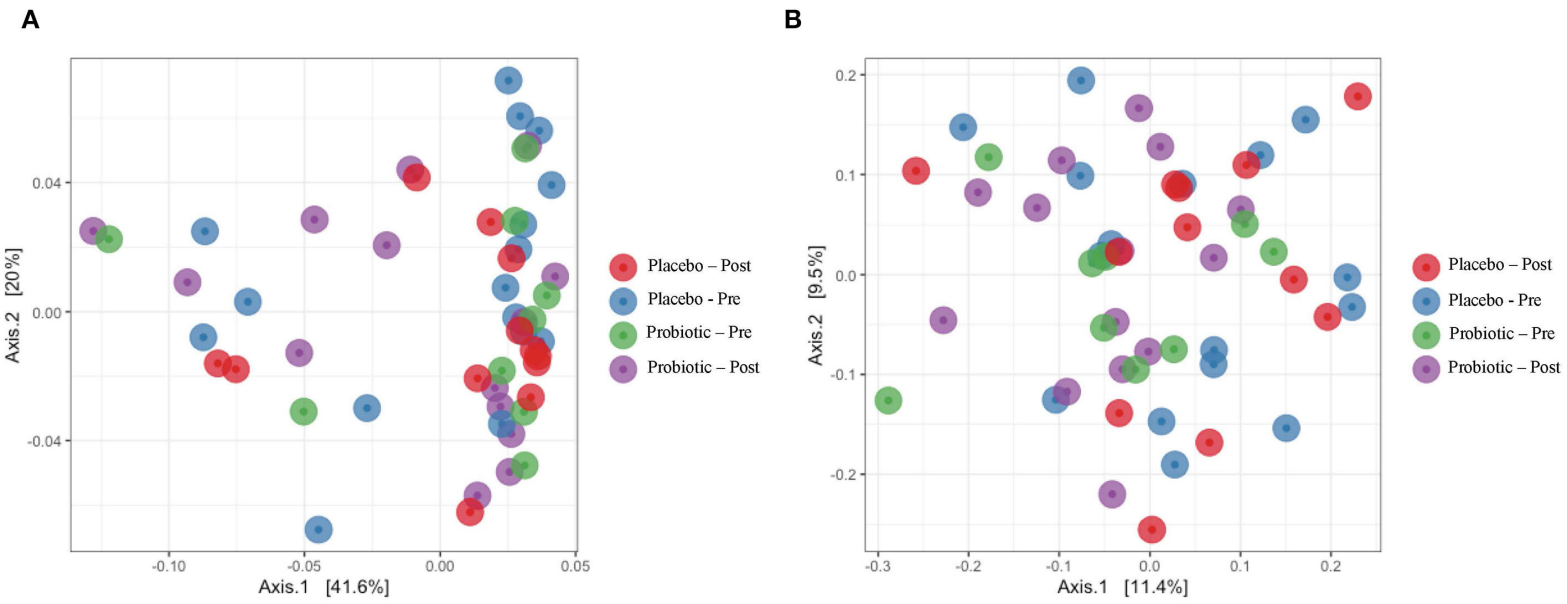
Principal coordinate plots with **(A)** weighted UniFrac and **(B)** unweighted UniFrac.

**Figure 5:**
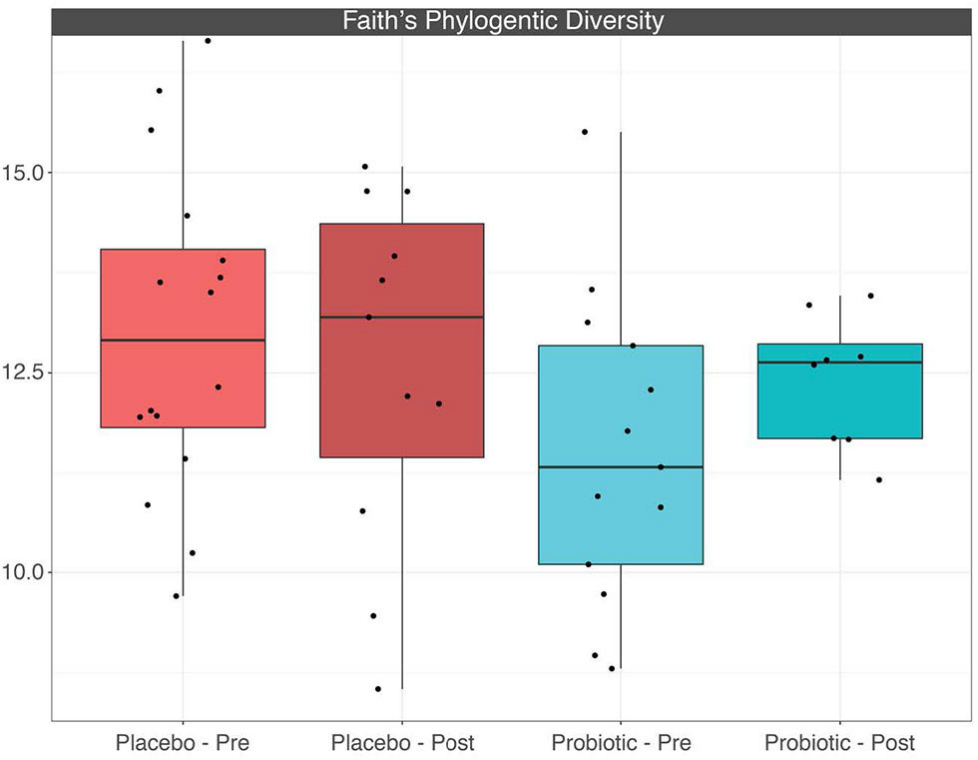
Boxplots depicting α-diversity based on Faith's phylogenetic diversity measure between treatment groups before and after placebo or probiotic administration.

**Figure 6:**
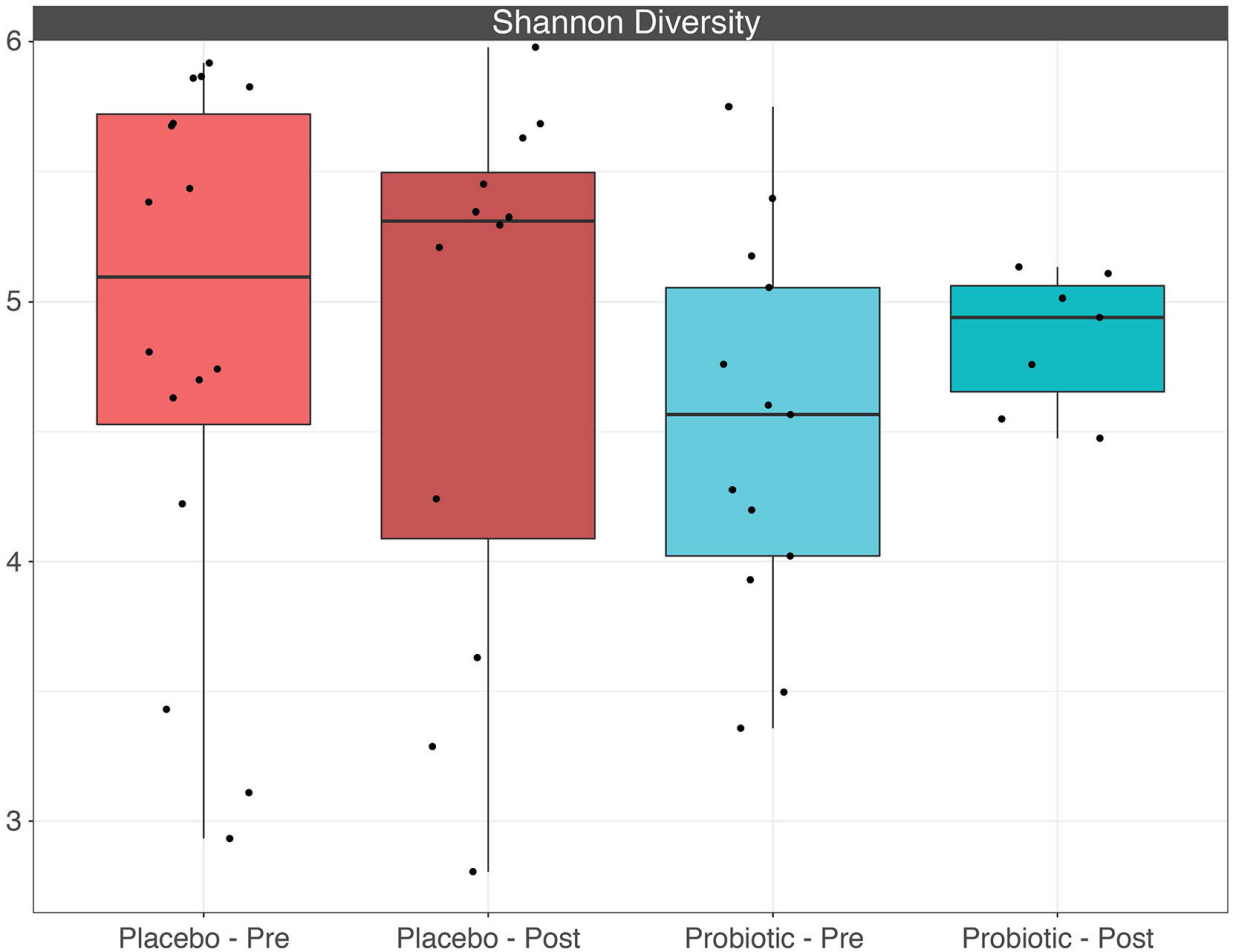
Boxplots depicting α-diversity based on Shannon diversity measure between treatment groups before and after placebo or probiotic administration.

The mean α-diversity for the study population was 108.8 for observed OTUs and 3.23 for Shannon diversity. Relative abundances of the most prevalent gut bacteria at the phylum level were 41.2% Firmicutes, 34.9% Bacteroidetes, and 17.9% Proteobacteria. The mean Firmicutes to Bacteroidetes ratio was 2.58 across the study, not significantly different across timepoints or treatment groups. *Bacteroides, Escherichia–Shigella, Faecalibacterium*, and *Prevotella* were the highest relative abundance genera across all samples. Overall, there were no differences between treatment group or timepoint in α-diversity, β-diversity, or taxa at the phylum or genus level (see Figures 3–6 for more details).

## Discussion

Findings from this trial support the feasibility, acceptability, and safety of supplementation with an anti-inflammatory/immunoregulatory probiotic, *L. reuteri* DSM 17938, among Veterans with PPC and PTSD symptoms. As noted above, we were broadly unable to detect differences between groups in biomarkers of inflammation (including IL-1α, IL-1β, IL-2, IL-6, IL-8, IL-10, IL-12p70, TNFα (active trimer), and IFNγ) or biomarkers of intestinal permeability. These findings are consistent with a recent meta-analysis, which found that probiotic administration among a wide-range of cohorts (e.g., healthy, IBS, obese, and TBI) had no significant effect on serum cytokines including IL-10 and TNFα (55).

Nonetheless, meta-analytic findings suggested that probiotic administration resulted in a reduction in serum CRP, with a weighted mean difference (WMD) of −1.35 mg/L [95% CI −2.15 to −0.55, *I*^2^ 65.1% (55)]. One of the articles noted in the meta-analysis was a randomized, single-blind study by Tan et al. of those with severe TBI (56). Those in the probiotic group received 0.5 × 10^8^ *Bifidobacterium longum* (ATCC 15697), 0.5 × 10^7^ *Lactobacillus bulgaricus* (ATCC 11842), and 0.5 × 10^7^ *Streptococcus thermophilus* (ATCC 19987). CRP levels were lower on days 15 and 21 among those who received the probiotic, relative to those in the placebo group.

In the current study, notable between-group differences were identified in terms of plasma concentrations of CRP, suggesting that CRP may be a viable inflammatory marker of interest. This may have been related to effects of *L. reuteri* DSM 17938 on the gut–liver axis (i.e., physiological links among gut dysbiosis, the integrity of the gut barrier and, via the hepatic portal vein, the hepatic immune and acute phase responses to gut-derived factors). This finding is also in line with previous studies suggesting that probiotic administration can decrease plasma CRP concentrations (55). Not all studies using *L. reuteri* DSM 17938 have observed decreases in plasma CRP concentrations, however (57, 58). Nonetheless, the potential for *L. reuteri* DSM 17938 to decrease plasma CRP concentrations may be of particular interest in the context of PTSD. In a Marine Resiliency Study of ~2,600 war zone-deployed soldiers, elevated plasma CRP concentrations at baseline predicted elevated PTSD risk 3 months post-deployment (18). To date, it remains unclear if interventions that decrease plasma CRP concentrations ameliorate PTSD symptoms when administered after the development of PTSD.

We also observed a significant impact of *L. reuteri* DSM 17938 on heart BPM during the TSST among Veterans with PPC symptoms and PTSD. Increased heart BPM during the TSST is a well-documented biomarker of the autonomic stress response. In this study, the TSST induced increases in heart BPM in participants who received placebo from ~75 to 95 BPM, with the peak response at the time of the math task. The results were virtually identical to the response observed in a study of 260 healthy non-smoking males aged 16–60 years (59). Individuals who had received daily supplementation with *L. reuteri* DSM 17938 responded with a significant reduction in the TSST-induced increase in BPM, relative to individuals who received placebo. Moreover, the observation that *L. reuteri* DSM 17938 had no effect on perceived stress suggests that the reduction in the TSST-induced tachycardia was not secondary to a reduction in perceived stress by the participants. Rather, *L. reuteri* DSM 17938 may alter autonomic output in a way that directly reduces stress-induced sympathetic outflow.

In support of this hypothesis, a recent study found that an 8-week administration of a fermented milk product with probiotic (FMPP) among healthy women (60) reduced reactivity in a widely distributed network of brain regions associated with responses to a validated negatively valenced emotional face attention task (60). Imaging data suggested a shift away from an arousal-based resting-state network and toward a regulatory network. The former network contained sensory regions including thalamus and insula, limbic regions including cingulate gyrus, amygdala, hippocampus, parahippocampal gyrus, the basal ganglia, and attention-related regions (BA 40) consistent with previous periaqueductal gray connectivity findings (61). Functional studies have demonstrated that the periaqueductal gray plays an important role in determining patterns of autonomic activity associated with different forms of emotional coping (62). Together, these data support the hypothesis that anti-inflammatory/immunoregulatory probiotic interventions alter patterns of autonomic activity responses during stress exposure, potentially through a shift away from an arousal-based resting-state network and toward a regulatory network.

Additionally, the use of *L. reuteri* DSM 17938 for 8 weeks did not significantly alter the composition of the gut microbiome. It is possible that alterations occurred in the participants' gut microbiomes that were not observed in the stool, as noted in an 11-strain probiotic administration study of mice and humans (63). Alternately, the use of *L. reuteri* DSM 17938 may have induced a transient and individual-specific response that was not detectable using the given sequencing technique (16S), or findings could be confounded by the relatively small sample size. In the present study, the probiotic was administered well after the occurrence of the mTBI. In animal models, it has been observed that the initial dysbiosis of the gut microbial community from injury might be limited to days post-injury (64). Therefore, it is possible that administration of a probiotic immediately following injury could have a more dramatic observable and lasting influence on gut microbiome composition. Findings associated with the lack of changes observed in the gut microbiome may also have been due to sampling procedures. Future trials should include sampling mid-supplementation (e.g., 4 weeks) in addition to post-supplementation.

## Limitations

Like all other studies, this one had a number of limitations. Efforts for this project were exploratory in nature, and one of the primary goals was to evaluate feasibility, acceptability, and safety. By intention, this initial study was not designed to evaluate changes in symptoms associated with clinical conditions. As such, the sample size was small, and many physiological outcome measures were obtained and analyzed. Funding was also limited, which restricted the number of samples that could be analyzed. Ideally, there would be at least one timepoint for all biological measures while the participants were on the probiotic or placebo to assess a longitudinal change.

## Conclusions

Taken together, results support the submission of a large, randomized, double blind, placebo-controlled trial focused on mechanistic links between biological markers and clinical outcomes, with the eventual goal of evaluating the efficacy of *L. reuteri* DSM 17938 for decreasing PPC and PTSD symptoms.

## Data Availability Statement

Department of Veterans Affairs policies will dictate the sharing of data. Interested parties should contact the corresponding author.

## Ethics Statement

The studies involving human participants were reviewed and approved by Colorado Multiple Institutional Review Board, as well as the local VA R&D Review Board. The patients/participants provided their written informed consent to participate in this study.

## Author Contributions

LB, JF, AH, CL, TP, and KS-Y conceptualized the research. KS-Y and CS provided data curation. JF, CS, and AH provided formal analysis, while LB acquired funding, project administration, and resources. KS-Y, MM, and HW conducted the investigations. LB, AH, and CL provided the methodology and JF the visualization. LB, DB, JF, AH, CL, MM, TP, CS, KS-Y, and HW wrote the original draft, while LB, DB, JF, AH, CL, MM, TP, CS, KS-Y, and HW reviewed and edited it. All of the authors contributed to this article.

## Conflict of Interest

LB consults for sports leagues. CL serves on the Scientific Advisory Board of Immodulon Therapeutics Ltd. The remaining authors declare that the research was conducted in the absence of any commercial or financial relationships that could be construed as a potential conflict of interest.
